# Obituary

**Published:** 1852-02

**Authors:** 


*Obituary.* — Died, on the 24th ult., at the Buffalo Hospital of the Sisters of Charity, Sister Symplicia, formerly Mary Ann Hughes, of Baltimore, aged 27 years.

Sister Symplicia was one of the band of pious females whose lives are devoted to the offices of christian charity. Of twelve Sisters who have been engaged in nursing the sick at the hospital in this city during the last three years, seven have contracted fever from fever patients received at the Institution. Six have, providentially, passed through the disease in safety. In the case of Sister Symplicia, death occurred on the tenth day of the febrile career.

